# The Anti-Proliferation, Cycle Arrest and Apoptotic Inducing Activity of Peperomin E on Prostate Cancer PC-3 Cell Line

**DOI:** 10.3390/molecules24081472

**Published:** 2019-04-15

**Authors:** Yunzhi Li, Jigang Pan, Meng Gou

**Affiliations:** 1 School of Pharmacy, Anhui University of Chinese Medicine, Hefei 230031, China; 2 School of Basic Medical Science, Guizhou Medical University, Guiyang 550025, China; hytss@126.com; 3 School of Life Sciences, Liaoning Normal University, Dalian 116081, China

**Keywords:** secolignan, peperomin E, cytotoxicity, apoptosis, cell cycle

## Abstract

Peperomin E is a natural secolignan existing distributed in the plants of the genus *Peperomia*. Previous investigations demonstrated that peperomin E showed potential antitumor activity in some cancer lines, but it is unclear whether peperomin E has an effect on prostate cancer cell lines. The aim of the present study is to investigate its effects on proliferation inhibition, apoptosis-inducing and cell-cycle arrest activity using a prostate cancer PC-3 cell line. The proliferation inhibition was evaluated by MTT assay, apoptosis was detected by Annexin V/propidium iodide (PI) staining and Hoechst 33258 staining, cell cycle distributions were measured by flow cytometry, and western blot analysis was used to determine specific cellular apoptotic protein expressions of Bcl-2, Bax, caspase-3 and cleaved-caspase-3. According to the results of this study, peperomin E exhibited significant anti-proliferation activity on PC-3 cell lines in vitro in a dose-dependent manner. Peperomin E treatments lead to marked morphological changes. Apoptotic cell count and cell-cycle distribution at G2/M phase significantly increased with increasing concentrations of peperomin E. The down-regulated expression level of Bcl-2 and up-regulated expression level of Bax and cleaved-caspase-3 compared with the controls were also observed after peperomin E treatment. These data suggest that peperomin E exhibited proliferation inhabitation, apoptosis-inducing and cell-cycle arrest activity on PC-3 cell lines. The anti-proliferation effect of peperomin E on PC-3 cells should result partly from its cell-cycle arrest and apoptosis-inducing activity, whereas the increasing of the Bax/Bcl-2 ratio and activation of caspases-3 play an important role in the development of apoptosis.

## 1. Introduction

The incidence of prostate cancer is the second highest among male malignant tumors in the world. According to statistics, there were number 1.4 million new cases worldwide in 2016, with 381,000 deaths that year [1]. In China, the incidence rate of prostate cancer has been increasing with the aging population and it is seriously harmful to male health [2]. Nowadays, androgen deprivation therapy (ADT) is the standard treatment for patients with prostate cancer, but, generally, after 12–24 months’ ADT in almost all prostate cancer cases will progress to castration-resistant prostate cancer (CRPC). In recent years, although some new drugs, such as docetaxel, cabazitaxel, the CYP17A1 inhibitor abiraterone, and the androgen receptor inhibitor enzalutamide, have been used as a first-line therapy to CRPC, they have a limited effect on disease outcomes. For example, cabazitaxel, which is a dimethoxy derivative of docetaxel, passes more easily through the blood-brain barrier of patients than docetaxel, but the overall and progression-free survival of patients with CRPC treated with cabazitaxel are only 15.77 and 5.52 months, respectively [3]. Therefore, it is important to find new drugs to treat prostate cancer.

**Figure 1:**
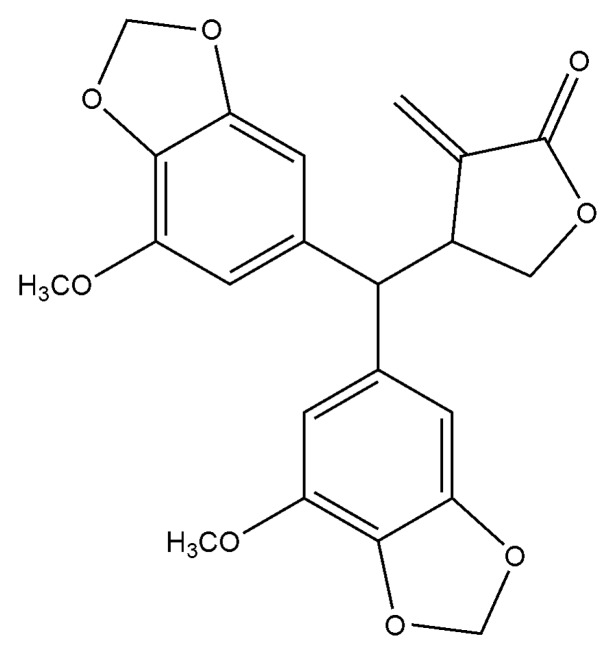
Chemical structure of peperomin E.

Secolignans are a rare kind of lignan, which originate from arylnaphthalene lignans by decomposition, oxidation and cyclization reactions. To date, only 32 compounds were obtained from three genera, i.e., *Peperomia*, *Urtica* L. and *Selaginella*, and most of them were obtained from *Peperomia* [4]. It has been found that secolignans have diverse bioactivities, such as anti-tumor [5], anti-inflammation [6], anti-HIV [7] and antifeedant properties [8]. To screen for bioactivite compounds from Chinese herbs, we carried out a phytochemical investigation into *Peperomia cavaleriei* and obtained a secolignan, i.e., peperomin E (Figure 1), which has been found in the same genus plants *Peperomia dindigulensis* [9], *Peperomia pellucida* [10] and *Peperomia sui* [11]. Previous investigations showed that peperomin E displayed significant proliferation inhibition activities in several cancer cell lines such as A549, Lovo, MCF-7, HL-60, VA-13 and HepG 2, HONE-1 and NUGC-3, etc. [5,10,11,12]. However, it remains unclear whether peperomin E has anyy effect on prostate cancer cell lines. The PC-3 cell line is a kind of androgen-independent prostate cancer cell, which is widely used as a cell model to study prostate cancer [13]. To screen underlying lead compounds, we investigated its proliferation inhibition, cell-cycle arrest and apoptosis-inducing activity in the prostate cancer PC-3 cell line as well as the preliminary mechanism of apoptosis inducement for the first time in this paper.

## 2. Results

### 2.1. Effects of Peperomin E on PC-3 Cell Proliferation

Proliferation-inhibition activity of peperomin E was examined in the prostate cancer PC-3 cell line using the MTT assay. The cells were separately treated with drugs at concentrations of 0, 1, 10, 20, 50, and 100 µg/mL for 24, 48, 72 h. 

**Figure 2:**
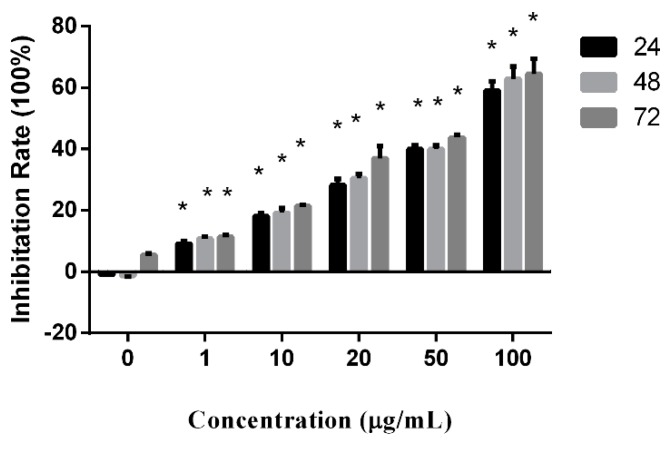
Effect of peperomin E on cell proliferation as determined by MTT assay. * *P* < 0.05.

As shown as Figure 2, the proliferation inhibition rates (IR) significantly increased along increased concentrations of the drug. Though the IR also increased to keep pace with prolonged treatment times, there was no statistical significance. According to the results, peperomin E exhibited a potent anti-proliferation activity in the PC-3 cell line in vitro in a dose-dependent manner. 

### 2.2. Effects of Peperomin E on Cell Apoptosis Inducement

**Figure 3:**
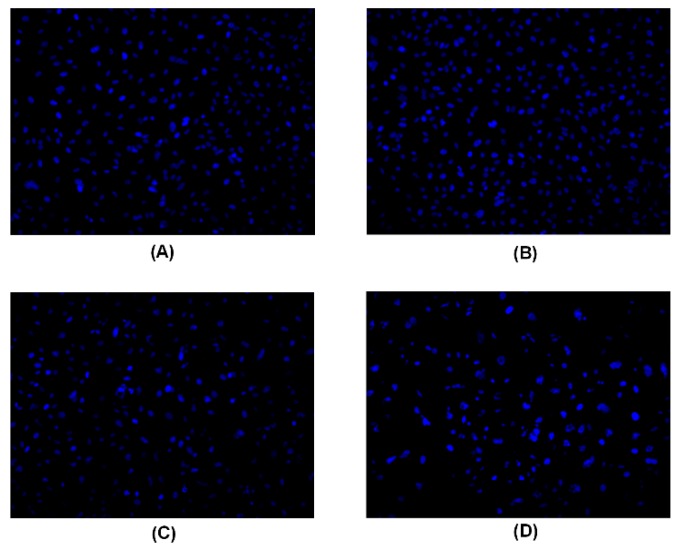
PC-3 cancer cells treated with peperomin E for 72 h with the indicated concentration by Hoechst 33258 staining. **A**, Control; **B**, 10 µg/mL peperomin E; **C**, 30 µg/mL peperomin E; **D**, 50 µg/mL peperomin E.

**Figure 4:**
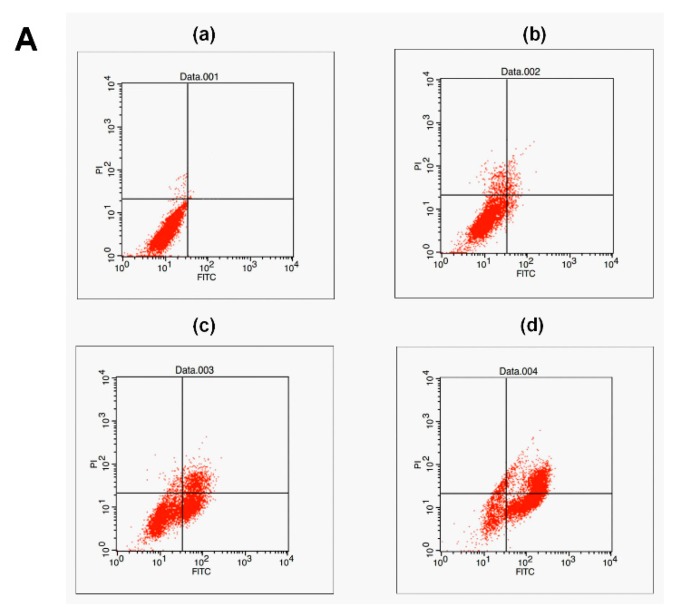

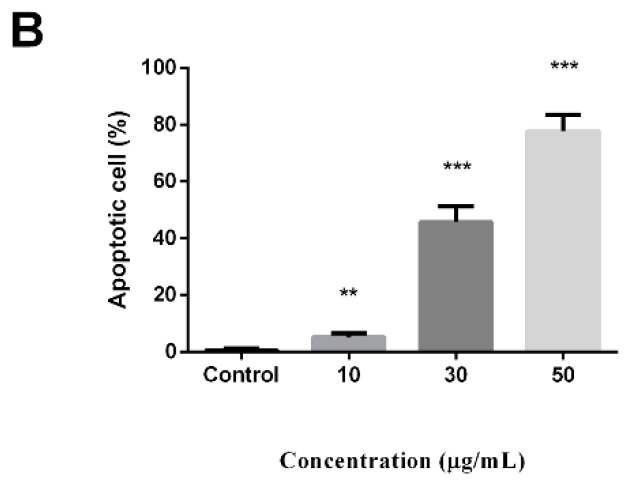
Effect of peperomin E on apoptosis of PC-3 cells by flow cytometry for Annexin-V-FITC and propidium iodide (PI) dual labeling. (**A**) Cells were treated with a vehicle (**a**) or peperomin E at 10 µg/mL (**b**), 30 µg/mL (**c**) or 50 µg/mL (**d**) for 72 h. The cell populations in the lower right represents early apoptotic cells, upper right represents late apoptotic cells. (**B**) The apoptotic rates of PC-3 cells induced by Pepromin E. Data are presented as means ± SD (*n* = 3). * *P* < 0.05, ** *P* < 0.01, *** *P* < 0.001 versus Con.

A Hoechst 33258 staining experiment was used to detect apoptotic cells. After treatment with peperomin E, some typical morphological changes relating to apoptosis were found in treated cells, such as nuclear shrinkage and chromatin fragmentation, whereas untreated cells were regular-shaped and stained evenly (Figure 3). This result suggests that peperomin E could induce apoptosis of PC-3 cells. To further verify its apoptotic activity and measure apoptosis rate, peperomin E was added to the cells with different concentrations for 72 h and analyzed by Annexin V and propidium iodide (PI) staining. The results are shown in Figure 4. When PC-3 cell lines were treated with 10, 30 and 50 µg/mL of peperomin E, ~5%, 45% and 78% of the cells were annexin V positive, respectively, whereas only ~0.1 % were annexin V positive in the control group. These data indicate that peperomin E could significantly induce apoptosis of PC-3 cells in a dose-dependent manner.

### 2.3. Effects of Peperomin E on PC-3 Cell-Cycle Distribution

**Figure 5:**
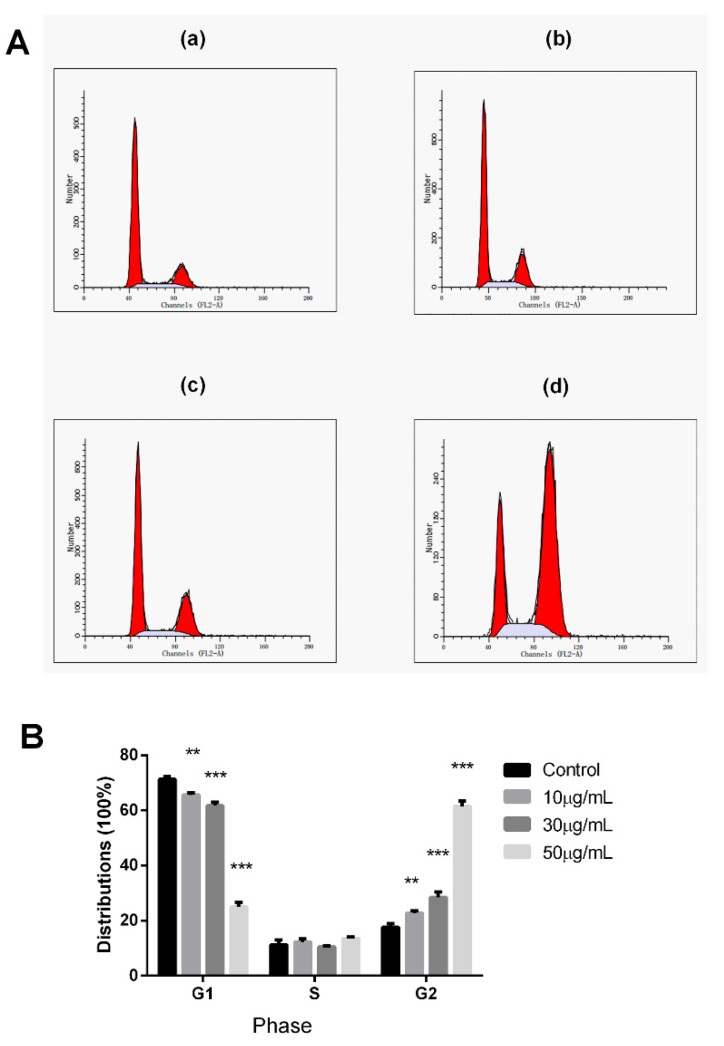
Analysis of cell cycle distributions. (**A**) PC-3 cancer cells were untreated (**a**) or treated with peperomin E (**b**, 10 µg/mL; **c**, 30 µg/mL; **d**, 50 µg/mL) for 72 h, then analyzed by flow cytometry. (**B**) The distribution and percentage of cells in G1, S and G2/M phase of the cell cycle are indicated. Data are presented as means ± SD (*n* = 3). * *P* < 0.05, ** *P* < 0.01, *** *P* < 0.001 versus Con.

Cell proliferation is well associated with the regulation of cell-cycle progression. Therefore, the effect of peperomin E on cell-cycle distributions were evaluated by flow cytometry in PI-stained cells in this experiment. As shown as Figure 5, after treatment for 72 h with peperomin E at a concentration of 50 μg/mL, cells in the G2/M distributions dramatically increased from 17.35% to 63.34% compared to the control cells, whereas the percentage of cells in the G1-phase dramatically decreased from 71.52% to 23.36%. The results demonstrate that peperomin E could significantly induce cell-cycle arrest at G2/M phase.

### 2.4. Effect of Peperomin E on the Expression Levels of Bax, Bcl-2, Caspase-3 and Cleaved-Caspase-3 in PC-3 Cells

As is known, the proteins in the Bcl-2 family and caspase family play critical roles in the apoptotic process. Thus, the expression levels of the important proteins involved in apoptosis in these two types of families were measured by western blot analysis. 

**Figure 6:**
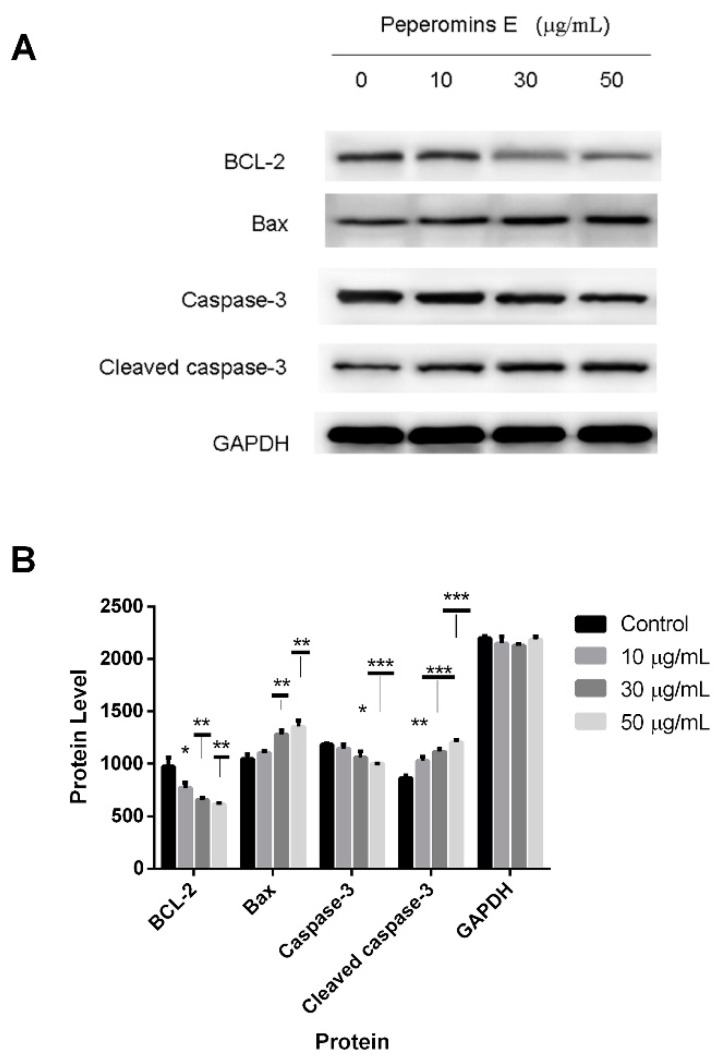
Effects of peperomin E on the protein levels of apoptosis related biomarkers in cells by western blotting. (**A**) The representative Western blot images showing the effects of peperomin E on the levels of Bax, Bcl-2, Caspase-3, cleaved-caspase-3 in PC-3 cells. GAPDH was used as the internal control (bottom panel). (**B**) Quantitation of significantly altered protein levels in PC-3 cell line by densitometry. Data are presented as means ± SD (*n* = 3). * *P* < 0.05, ** *P* < 0.01, *** *P* < 0.001 versus Con.

The results are shown in Figure 6. After treating cells with increasing concentrations (0, 10, 30 and 50 µg/mL) of peperomin E for 72 h, the expression levels of Bax and cleaved-caspase-3 significantly increased, whereas the expression levels of Bcl-2 and caspase-3 significantly decreased in the PC-3 cells in a dose-dependent manner. These data suggest that peperomin E could up-regulate the ratio of Bax/Bcl-2 and activate caspase-3 in the PC-3 cell line.

## 3. Discussion

Natural products are an important source of clinical anti-tumor agents. Natural lignans possess diverse chemical structures and bioactivities, and have attracted more and more attention from researchers. Previous investigations demonstrated that the genus *Peperomia* is rich in various types of lignans, and secolignan is a type of novel lignan found in *Peperomia* for the first time [14]. Peperomin E is a natural secolignan (Figure 1), which is widely distributed in *Peperomia*. Previous studies demonstrated that peperomin E was a potent cytotoxic agent against a variety of cancer cell lines. For example, the half inhibitory concentrations for HepG2, MCF-7, HL-60 and Hela cell lines are 12.1, 3.9, 1.8, and 11.1 μM, respectively [5,10]. A recent investigation showed that peperomin E had anti-proliferation, apoptosis-promoting and cell-cycle arrest activities in non-small-cell lung cancer cell lines, and the underlying mechanism was partly attributable its ability to demethylate and reactivate methylation-silenced tumor suppresser genes through direct inhibition of the activity and expression of DNA methyltransferase [15]. For screening the lead compound of anti-prostate cancer, we investigated its bioactivity on prostate cancer PC-3 cell lines in this study for the first time, and found that peperomin E could inhibit the proliferation of human prostate cancer PC-3 cells in a dose-dependent manner. Hoechst 33258 staining revealed that the drug could evoke apoptosis, and this was further evidenced by Annexin V and PI staining. Western blot analysis showed that the ratio of Bax/Bcl-2 was up-regulated and caspase-3 was activated after treatment with peperomin E. In addition, cell-cycle arrest at G2/M phase was also revealed according to the data of flow cytometry, which is different to peperomin E on non-small-cell lung cancer cell lines which cell-cycle arrest at G1/S phase [15].

It is well known that apoptosis is a programmed cell death different to necrosis. Dysregulation of apoptosis leads to pathological conditions including cancer and autoimmune diseases. Therefore, researchers have focused efforts on the development of strategies designed to selectively induce apoptosis in cancer cells [16]. It has proved that Bcl-2 family proteins were critical regulators of the apoptotic pathway [17]. Among members of the Bcl-2 family, Bax is a pro-apoptotic protein, whereas Bcl-2 is a potent suppressor of apoptosis. As Bax and Bcl-2 have opposing functions, the ratio of Bcl-2 to Bax is an important index to evaluate cell apoptosis. Higher levels of Bax relative to Bcl-2 after a death signal may increase the cells’ susceptibility to apoptosis [18,19]. In this study, after treatment with peperomin E, the up-regulated Bax and down-regulated Bcl-2 result in an increasing ratio of Bax to Bcl-2, revealing that a Bcl-2-related signaling pathway was involved in the apoptosis of PC-3 cells.

Caspases are a family of cysteine proteases and are one of the main executors of the apoptotic process or programmed cell death, and lead to characteristic morphological changes of the cell undergoing apoptosis, such as shrinkage, chromatin condensation, and DNA fragmentation [20]. Among these apoptotic caspases, caspase-3 is one of the key executioners of apoptosis [21]. Both of the two major apoptotic pathways (death receptors and the mitochondrial pathway) are common to the activation of caspase-3 [22]. Therefore, we further examined the effects of peperomin E on the expression levels of caspase-3 and cleaved-caspase-3 by western blot analysis. As expected, the expression of the caspase-3 decreased and cleaved-caspase-3 significantly increased compared with controls. 

The cell cycle includes a number of checkpoints that allow the cell to repair its damaged DNA. Checkpoints at G1/S and G2/M transitions are essential regulatory gates during cell-cycle progression, whereas loss of cell-cycle checkpoints ahead of completing DNA repair can activate the apoptotic cascade and result in cell death [23,24]. Therefore, there is no doubt that a cell-cycle target-based drug will be an excellent source of new anticancer compounds [25]. In this study, after treatment with peperomin E, the cell-cycle distributions were significantly accumulated at the G2/M phase, demonstrating that peperomin E could induce cell-cycle arrest on PC-3 cells. This might be part of the reason why peperomin E results in anti-proliferation and apoptosis-inducing activity in PC-3 cell lines. 

In conclusion, the results indicate that peperomin E could inhibit cell proliferation, inducing apoptosis and cell-cycle arrest at the G2/M phase in vitro in the PC-3 cell model. Proliferation inhibition was associated partly with the inducement of an apoptotic effect and cell-cycle arrest, whereas apoptosis was associated with up-regulation of the ratio of Bax/Bcl-2 and activation of caspase-3. 

## 4. Materials and Methods

### 4.1. Chemicals 

Peperomin E, whose chemical structure is shown in Figure 1, was isolated from the *Peperomia* plants. It was dissolved in DMSO to make stock solutions (100 µg /mL) and stored at −20 °C. Before the experiment, it was diluted in cell culture medium to the indicated concentration. 

### 4.2. Cell Culture

The PC-3 cell line (human androgen independent prostate cancer) were obtained from BeNa Culture Collection Biotechnology Co. Ltd. (Beijing, China). The cells were maintained in RPMI-1640 cultured medium (Hyclone, Logan, UT, USA) supplemented with 10% fetal bovine serum (FBS, Gibco-BRL, Gaithersburg, MD, USA). Cultures were maintained at 37 °C in a CO_2_ incubator with a controlled humidified atmosphere composed of 95% air and 5% CO_2_. 

### 4.3. Cell Proliferation Assay

In order to investigate the anti-proliferation activity of peperomin E on PC-3 cell line in vitro, cell viability was evaluated by MTT assay. Briefly, cells were seeded in 96-well plants at 1 × 10^3^ − 5 × 10^3^ per well, and cultured at 37 °C overnight, then treated with peperomin E at different concentrations (1, 10, 20, 50, or 100 μg/mL) in a total volume of 100 µL media containing 10% PBS, and untreated cells were utilized as controls. After incubation for different times (24, 48, 72 h), 110 µL Formazan solution was added to each well and shaken for 10 min at low speed for dissolving crystals. The optical density was measured using a microplate spectrophotometer (Bio-Tek, Winooski, VT, USA) at 490 nm. The IC_50_ values were the drug concentrations causing 50% inhibition of cell growth. Doxorubicin was used as positive control.

### 4.4. Hoechest 33258 Staining Assay

Hoechst 33258 staining is used to distinguish condensed nuclei in apoptotic cells. Cells were treated with different concentrations of peperomin E for 72 h, fixed by 4% polyformaldehyde for 10 min, then the fixative was discarded, and washed with PBS twice. Next, the fixed cells were stained with Hoechst 33258 (5 µg/mL in PBS) for 5 min, washed by PBS five times, and observed under a fluorescence microscope (Olympus, Tokyo, Japan).

### 4.5. Annexin V and PI Staining for Apoptosis Detection

The effect of peperomin E on the apoptosis inducement of cells was further determined by Annexin V-FITC/PI staining. After incubating with drug or vehicle control (0.45% DMSO) for 72 h, the cells were harvested, washed with cold PBS three times, and resuspended in 200 μL binding buffer. Then, 10 μL Annexin-V-FITC and 10 μL PI were added, incubated for 30 min at 4 °C in the dark, and analyzed by flow cytometry (Becton Dickinson, Franklin Lakes, NJ, USA).

### 4.6. Flow Cytometric Analysis of DNA Cell Cycle

Cell-cycle analysis was performed by flow cytometry (FACS, FACS Calibur, Becton Dickinson) after cellular staining with PI. Cells were treated by peperomin E with different concentrations, separately. After culturing for 72 h, the cells were washed with PBS twice, fixed in 70% ethanol at 4 °C overnight. Then, the cells were centrifuged and washed with PBS twice, stained with PI at a final concentration of 50 mg/mL and RNase at a final concentration of 50 mg/mL, incubated at 37 °C for 30 min, then analyzed by FACS (Becton Dickinson).

### 4.7. Western Blot Analysis 

After being treated with peperomin E for 72 h, cells were washed with cold PBS twice, adding lysis buffer containing protease and phosphatase inhibitors and lysis cells altogether at 4 °C, then cells were heated for 10 min at 95 °C, and centrifuged at 12,000× *g* for 10 min at 4 °C. Next, the protein of the clear supernatant was collected and frozen at −80 °C. Its concentrations were quantified using a BCA protein assay kit (Thermo Fisher Scientific, Waltham, MA, USA). The protein lysate was separated by electrophoresis in a 12% sodium dodecyl sulfate polyacrylamide gel electrophoresis (SDS-PAGE) and transferred to a polyvinylidene fluoride (PVDF) membrane (Millipore, Bedford, MA, USA), then was blocked for 2 h in blocking buffer (5% skimmed milk) at room temperature, washed three times in PBST (PBS contained 0.05% Tween-20). The membranes were incubated with the relevant antibodies overnight at 4 °C. After washing with PBST buffer three times, the protein bands were detected with ECL substrate solution and detected by an automated chemiluminescence immunoassay analyzer (Tanon-5200, Shanghai, China).

### 4.8. Statistical Analysis

Data are represented as mean ± SD of values according to three independent experiments. Statistical analyses were carried out using Student’s *t*-test. A P value of less than 0.05 was considered statistically significant.
